# Strategies for automatic generation of information processing pathway maps

**DOI:** 10.3389/fnimg.2025.1608390

**Published:** 2025-11-25

**Authors:** Anirudh Lakra, Cai Wingfield, Chao Zhang, Andrew Thwaites

**Affiliations:** 1 Department of Computer Science, University College London, London, United Kingdom; 2 MRC Cognition and Brain Sciences Unit, Cambridge, United Kingdom; 3 Institute for Data and AI, University of Birmingham, Birmingham, United Kingdom; 4 Department for Speech Hearing and Phonetic Sciences, University College London, London, United Kingdom; 5 Department of Electronic Engineering, Tsinghua University, Beijing, China; 6 Shanghai Artificial Intelligence Laboratory, Shanghai, China; 7 Beijing National Research Center for Information Science and Technology, Beijing, China

**Keywords:** magnetoencepalography, electroencaphlography, auditory processing, motion processing, information processing pathway maps

## Abstract

Information Processing Pathway Maps (IPPMs) are a concise way to represent the evidence for the transformation of information as it travels around the brain. However, their construction currently relies on hand-drawn maps from electrophysical recordings such as magnetoencephalography (MEG) and electroencephalography (EEG). This is both inefficient and contains an element of subjectivity. A better approach would be to automatically generate IPPMs from the data and objectively evaluate their accuracy. In this work, we propose a range of possible strategies and compare them to select the best. To this end, we (a) provide a test dataset against which automatic IPPM creation procedures can be evaluated; (b) suggest two novel evaluation metrics—*causality violation* and *transform recall*—from which these proposed procedures can be evaluated; (c) conduct a simulation study to evaluate how well ground-truth IPPMs can be recovered using the automatic procedure; and (d) propose and evaluate a selection of different IPPM creation procedures. Our results suggest that the *max pooling* approach gives the best results on these metrics. We conclude with a discussion of the limitations of this framework, and possible future directions.

## 1 Introduction

Functional brain mapping is the data-driven process of associating specific brain regions with critical functions such as vision, sensation, movement, and language. Gaining a clear understanding of where and how the brain performs these functions has wide-ranging implications. Accurate functional mapping, for instance, is essential for advancing neurotechnology performance and safety, as well as making neurosurgery more precise and reliable. Furthermore, it lays the foundation for exploring higher-order cognitive functions like memory, attention, and learning. Consequently, functional brain mapping remains a key focus in current research.

**Figure 1:**
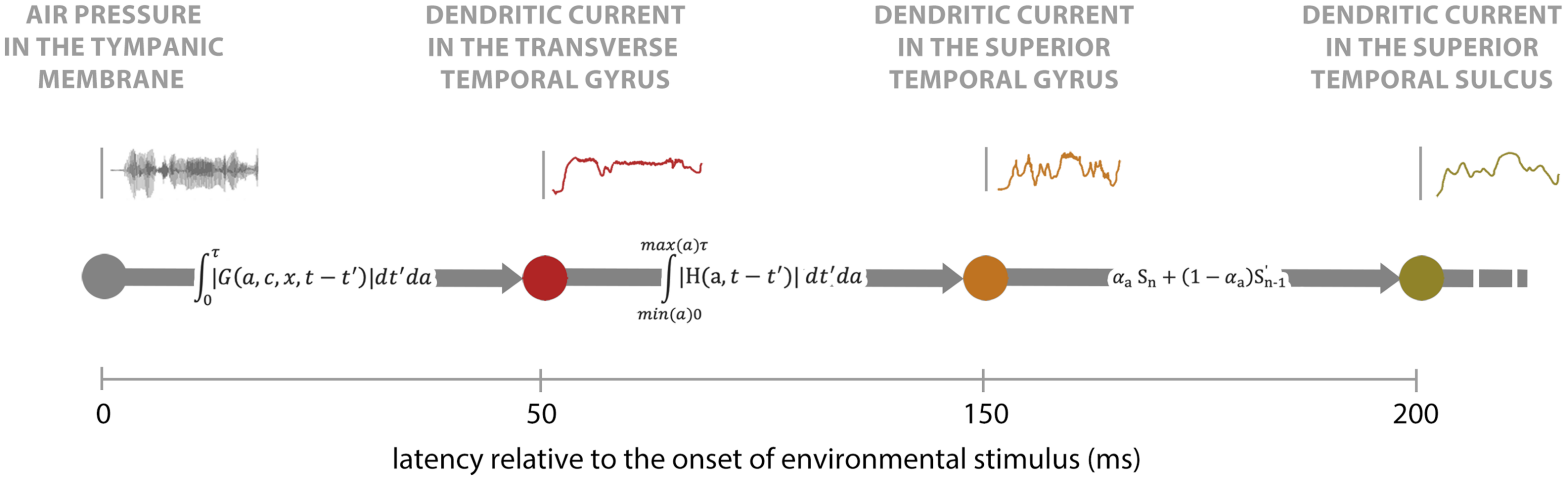
Example IPPM. Sound hitting the tympanic membrane undergoes a sequence of mathematical transformations as it travels up the auditory pathway, with the outputs of these transformations being entrained to neuronal activity in different locations in the nervous system and cortex. The position of each node on the x-axis denotes the latency at which the outputs of these transforms are entrained. Adapted from Thwaites et al. (2025).

A recent development in this field is the creation of *Information Processing Pathway Maps* (IPPMs) from electrophysiological neural recordings (Thwaites et al., 2025). IPPMs represent the sequences of mathematical transformations that describe how sensory information is processed as it travels through the nervous system and cortex (Figure 1). These maps have significant potential across various applications, including Brain–Computer Interfaces (BCIs), human prosthetics, and clinical interventions. To date, IPPMs have been developed for processes such as loudness processing (Thwaites et al., 2015, 2017), color processing (Thwaites et al., 2018), visual motion processing (Wingfield et al., 2025), and tactile processing (Thwaites et al., 2025).

Before discussing the construction of IPPMs, it is important to define some key terms. A *transform* refers to any mathematical function that hypothesizes how stimulus properties correspond to measured cortical activity in the human brain. Given the inherent spatial accuracy of our neuroimaging methodology, we resolve results into small, tessellating hexagons around 3 mm in diameter, referred to as *hexels*. *Expression* refers to the situation where the output of a particular transform correlates with the observed cortical activity in a specific hexel.

**Figure 2:**
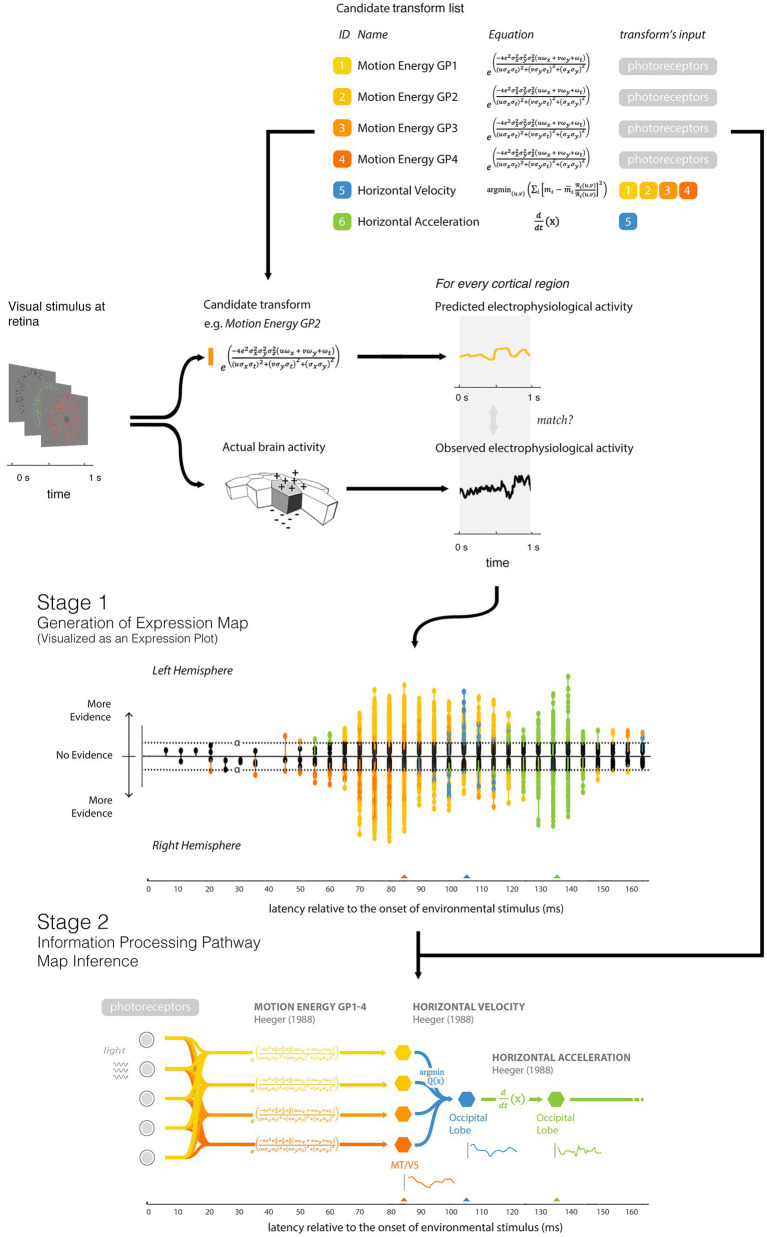
The main steps involved in IPPM creation. Figure and caption reproduced from Thwaites et al. (2025). In Stage 1, a list of candidate transforms is first hypothesized; in this example, six transforms related to motion processing were drawn from the optic-flow model of Heeger (1988) (shown in the Candidate Transform List). The electrophysiological activity of the brain in response to a given stimulus (measured using M/EEG) is matched to the pattern of neural activity predicted by each of the transforms being evaluated. Predicted and observed activity are tested for similarity and the resulting statistical parametric map displays the regions (hexels) where the match is statistically significant. This expression map is here visualized as an expression plot; the stems show the latency at which each of the hexels for each hemisphere best match the output of the tested transform, with the y-axis shows the evidence supporting the match at this latency. By convention, both the left and the right hemispheres plots are mirrored around the x-axis for ease of comparison, with ‘no evidence’ at the midline, and hexels with higher evidence plotted further away from the midline. If any of the hexels have evidence, at their best latency, this is indicated by evidence higher than a predefined threshold which has been adjusted to take account of multiple comparison correction. For example, in the approach of Thwaites et al. (2017, 2018), the y-axes correspond to *p*-values, calculated through a one-tailed two-sample t-test between a distribution of the observed correlation of the hexel activity with the candidate transform and a suitable null distribution. The stems are colored according to the transform. In Stage 2, IPPMs are inferred from the expression map and the candidate transform list. This second stage is currently done by hand. The final IPPM describes the transforms underlying human visual motion processing (Wingfield et al., 2025).

IPPMs can be created using any time-varying measure of neural activity, including electroencephalographic (EEG) and magnetoencephalographic (MEG) recordings. The process involves two main stages (Figure 2).

In Stage 1, the researcher starts with time-varying neural activity data from a large number of hexels, recorded while participants engaged in tasks like listening to a podcast or watching a movie. The researcher also begins with a *candidate transform list* (CTL) which includes potential transforms believed to occur in the nervous system. The stimulus is processed through each transform in the list, generating precise predictions of cortical activity. These predictions are then compared to the actual neural activity across various latencies. After passing through a model-selection procedure (Thwaites et al., 2017), a transform *expression map* is created. This result is often visualized as an *expression plot* (see Stage 1 of Figure 2, for example).

During Stage 1, the tested transforms are *input-stream-to-hexel* transforms, representing the relationship between the input stream (e.g., auditory or visual stimuli) and hexel activations. By contrast, IPPMs are constructed using *hexel-to-hexel* transforms, representing the relationships between different nodes within the IPPM. While Stage 1 does not explicitly test these *hexel-to-hexel* transforms, they can be inferred from the definitions in the CTL, and in Stage 2, the researcher uses these definitions, along with the expression data, to infer the IPPM.

**Figure 3:**
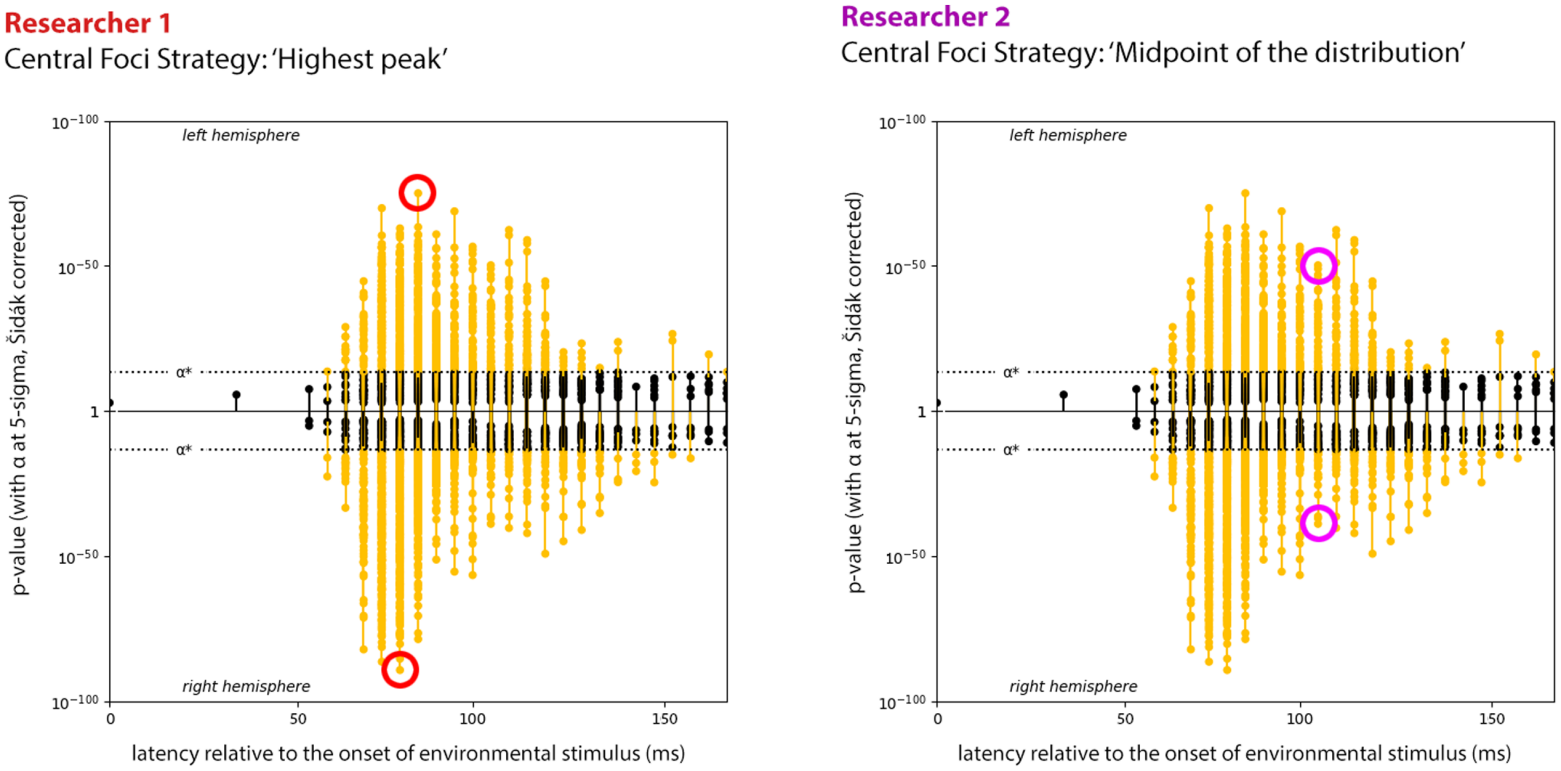
Example of two researchers using different strategies for finding the central foci of an expression cluster. Both plots show the left and right hemisphere expression for ‘Heeger Motion Energy GP2’. Due to inherent measurement error in EMEG recordings, the latency for neighboring hexels’ expression (characterized by the stems in the figure) are noisy, leading to characteristic ‘clusters’ of expression around a central peak. Different strategies for choosing the clusters’ centers can result in different interpretation of the results: the researcher on the left (red) is making their choice based on the highest peak in each hemisphere, while the researcher on the right (purple) is using the midpoint of the total spread of the cluster. Choosing different centers will lead to differences in subsequent IPPM structure. Adapted from Wingfield et al. (2025).

Despite the critical role of Stage 2 in IPPM creation, it can currently only be performed by visual inspection. This is due to the “blurred” nature of expression data, resulting from the inherent difficulty in source localization in EEG and MEG (Grave de Peralta-Menendez et al., 1996; Grave de Peralta-Menendez and Gonzalez-Andino, 1998). This difficulty leads to a phenomenon known as *point spread*, where responses bleed into neighboring cortical sources (Hauk et al., 2011). Consequently, hundreds of hexels may appear significantly entrained to given transforms, creating clusters of expression points in the expression plot. Resolving these points into separable effects can be challenging. Where one researcher might interpret them as a single focused effect and select the most significant point as representative, another may see two temporally overlapping effects (Figure 3). Distinguishing between a single temporally distributed effect and multiple shorter effects with some overlap requires the researcher’s judgment, guided by prior knowledge from the literature.

While IPPMs are effective tools for data visualization, inferring their structure through visual inspection of the expression plots has several inherent drawbacks. First, the process is subjective: there are no formalized rules for IPPM generation, which can lead to variations between researchers. Second, this subjectivity might tempt researchers to interpret the expression plot in ways that align with their study’s goals. Third, being forced to rely on prior literature to determine where distinct effects “should be” detracts from the objectivity of the process. Finally, inferring IPPMs though visual inspection of the expression plots can be labor-intensive, especially when mapping a large number of transforms. For IPPMs to be effectively used in clinical, diagnostic, or BCI contexts (Thwaites et al., 2025), they need to be fully data driven.

The structure of this paper is as follows. We formalize the problem in Section 3, giving a characterization of IPPM graphs as mathematical objects. In Section 4, we establish a framework for evaluating automatic-IPPM-generation strategies. We introduce two metrics for evaluating an IPPM and describe how they should be interpreted. In Section 5, we introduce a selection of strategies for automatically generating IPPMs, using a suite of denoising algorithms, and describe how they would be applied to EMEG data. We evaluate each of these strategies in two ways, with results presented in Section 6. First, we evaluate using a synthetic-data simulation where the ground-truth IPPM is known. Next, we evaluate on real-world EMEG data, using the evaluation to select an optimal strategy for automatically generating IPPMs. Finally, we discuss and summarize the analysis in Section 7.

In summary, this paper develops an automated IPPM generation system that implements the logic of an objective researcher. To evaluate this system, we propose a framework using two IPPM baselines—auditory loudness processing and visual motion processing—along with two metrics: *Causality Violation* (CV) and *Transform Recall* (TR). While handcrafted IPPMs might serve as a plausible gold standard, we avoid using them as benchmarks due to the risk of inherent errors. Instead, we focus on evaluation metrics that assess qualities necessary for a true IPPM. This framework aims to set the stage for future advancements in automatic IPPM generation.

## 2 Problem formalization

**Figure 4:**
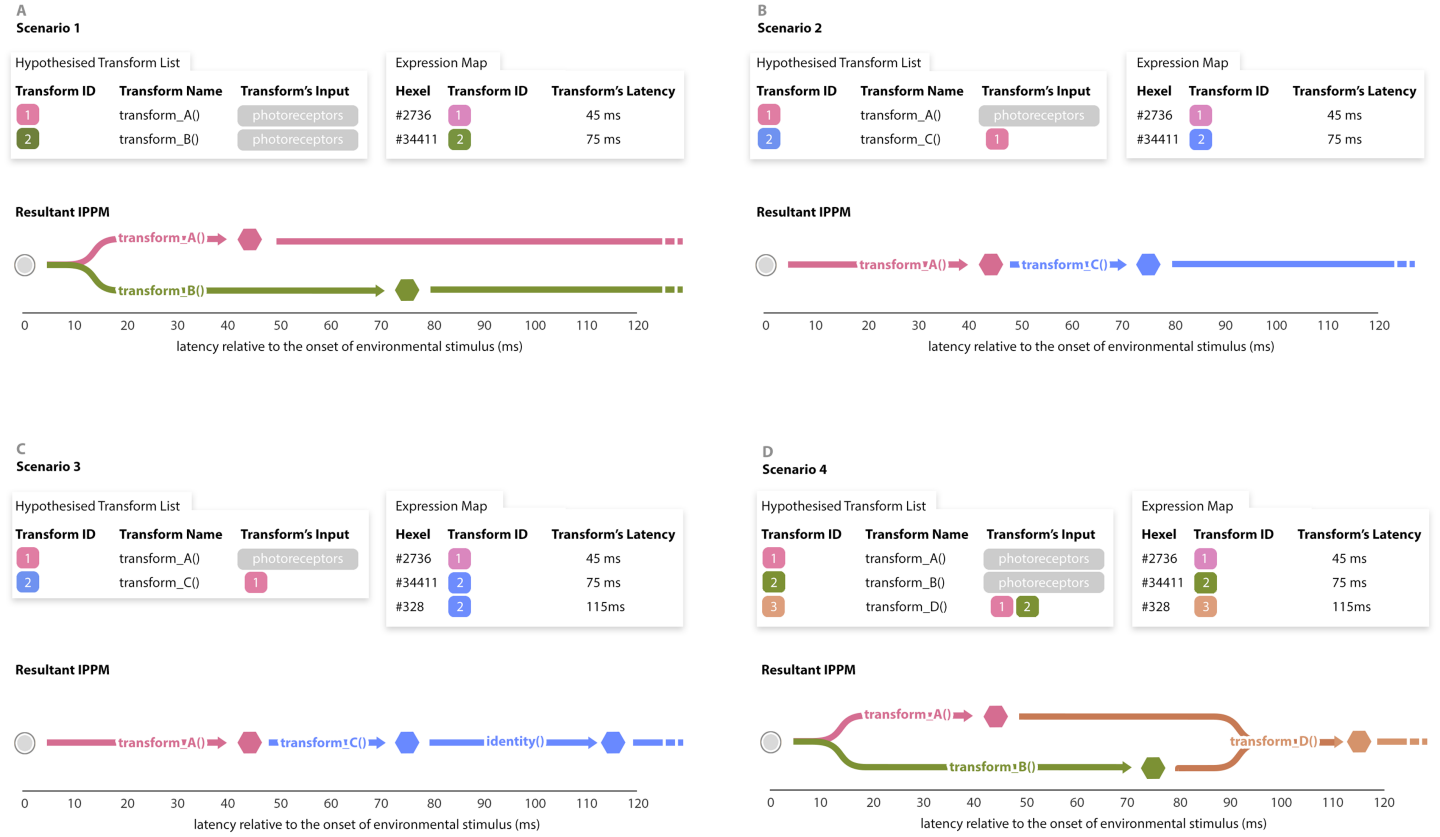
Some of scenarios that the IPPM generator might face. A The candidate transform list (CTL) allows us to convert from input-to-node transforms (present in the expression map) to node-to-node transforms represented in the resultant IPPM. **(A)** In this example, all transformations in the CTL are input-to-node, resulting in the above estimation of the IPPM. **(B)** In this scenario*, transform_B()* has been replaced with *transform_C(),* which has the output *transform_A()* as its input. This alters the resultant IPPM accordingly, placing the new transform in a sequence. **(C)** This scenario is the same as Scenario 2, except that the expression map has a second hexel that is entrained to the output of *transform_C().* This alters the resultant IPPM, adding an ‘empty’ transform which copies the information (unchanged) to another hexel. This is commonly known as the *null()* or *identity()* transform. **(D)** This scenario is the same as Scenario 1, except that the CTL has a third transform D which accepts the output of A and B as input, and the expression map has a hexel that is entrained to it. This alters IPPM, creating a sequence of three transforms between hexels. This list of scenarios is not exhaustive but aims to give a sense of the situations an automatic IPPM generator needs to be able to handle.

An IPPM can formally be defined as a Directed Acyclic Graphs (DAGs) whose nodes are significant hexels, and whose edges connect serially composed transforms. Given (1) expression data and (2) a candidate transform list (CTL) that includes information about the relationship between *input-stream-to-hexel* transforms and *hexel-to-hexel* transforms, we wish to create a DAG with these qualities. In particular, this information allows us to make inferences about whether transforms are taking place serially or in parallel (Figure 4).

In order to estimate which clusters of significant points derive from the same underlying effect, we apply a temporal clustering subsystem. The output of the clusterer is a much smaller set of significant hexels representing the temporal foci of separable effects. Given the clustered hexels and latencies, the graph generator then applies a candidate transform list to generate the required graph.

## 3 IPPM evaluation framework

As noted, handcrafted IPPMs are not a suitable comparator for the evaluation of automatic IPPM generation, due to the inherent subjectivity present in their production. But there are other ways to evaluate IPPM accuracy. In particular, all accurate IPPMs have fundamental properties about them that must be true, and we can assess an automated IPPM’s accuracy by estimating to what extent they comply with these properties.

We propose two such metrics. The first is Causality Violation (CV), which is based on the premise that information cannot travel backwards in time. Suppose we have a transform, *B*(·), which has the parent transform, *A*(·). Such a relationship suggests that information should travel forwards in time from parent to child, from *A*(·) to *B*(·). In the generated IPPM, this would be reflected by the nodes for A being placed earlier to those for B, with the arrows of causation leading from A to B. We define CV as the number of edges facing backwards divided by the number of edges in total. The IPPM map can be represented as *M = (V, E)* where V denotes the set of nodes, E denotes the set of directed edges, and *l(u)* denotes the latency of node *u*:


$$\mathrm{CV}=\frac{|\{(u,v)\in E\;|\;l(v)<l(u)\}|}{|E|}$$

Our second metric, Transform Recall (TR), focuses on ensuring that the IPPM retains as much useful information as possible. TR is defined as the proportion of detectable transforms in the candidate transform list that appear in the generated IPPM (where a detectable transform is one that shows significant evidence in the expression plot). This approach was motivated by the observation that an automatic IPPM generator cannot detect a transform missing from the expression data, so we should not penalize a candidate generator for missing it. Let *T*_D_ denote the set of transforms that appear in the denoised expression data and *T*_C_ denote the set of transforms that appear in the CTL:


$$\mathrm{TR}=\frac{|T_{D}|}{|T_{C}|}$$

CV and TR are, to some extent, complementary; each measures a different aspect of IPPMs that are often in tension with one another. While CV focuses on the correctness of the location of the nodes, TR evaluates the system sensitivity. By reducing the number of nodes, CV will tend to improve, since with less nodes it becomes less likely for a node to precede its parent, leading to less violations. However, this comes at the expense of TR as if the threshold for a cluster is too high, we can mislabel significant points, resulting in discarded nodes. Thus, CV prioritizes correct IPPMs with a minimal complexity while TR prioritizes IPPMs that capture the greatest quantity of salient information from the expression plot. Through both metrics, we can locate the model with the optimal fit but also with the greatest parsimony.

Poor CV or TR may arise from either an erroneous expression plot, inaccurate or incomplete CTL, or faulty clustering algorithm. In this paper, we are concerned with the last source of error—the clustering error. To identify the ideal generator, we need to isolate the clustering error, which requires controlling for other sources of error. One can achieve this by fixing a CTL established by prior literature. We analyze this assumption and its consequences in greater detail later in the Discussion section.

In practice, the proposed strategies require the estimation of suitable hyperparameters. Since CV and TR are in tension with one another, we select the hyperparameters by performing a grid-search over a range of values, plotting the Pareto frontier, and identifying points that first maximize TR, then CV. Our rationale for prioritizing TR is that it signifies that all the salient information in the expression plot has been captured, so all information required to create the perfect IPPM is there but was not attained due to one of the sources of error.

## 4 Strategies for automatic IPPM generation

In addition to setting out an IPPM-accuracy framework, we test a range of solutions that aim to automatically generate IPPMs. Automatic IPPM generation requires two steps, *Clustering*, which attempts to negate the effects of point spread, and *The IPPM Builder*, which creates a suitable DAG from the CTL and the clustered expression map. The rules that underpin the second stage IPPM Builder can become quite complex but are relatively straightforward to justify (see section 5.2). The biggest issue in accurate IPPM generation is the initial clustering step, which can make a big difference to the accuracy of the final IPPM. As a result, we have chosen to use the same IPPM Builder rules with all clustering strategies tested in this paper.

### 4.1 Clustering

As noted, EMEG source reconstruction inevitably blurs cortical activity. ‘Clustering’, or ‘denoising’, refers to compensating for this signal–noise mixing, which arises both from the inherent spatial imprecision of EEG/MEG and from additional experimental noise (e.g., non-cortical artifacts). Two main approaches are commonly distinguished: *spatial denoising*, which aims to correct for spatial blurring in a manner analogous to deblurring an image, and *temporal denoising*, which seeks to mitigate errors in the timing of EEG/MEG signals.

In this study, we focus exclusively on temporal denoising. Spatial point sources may be mislocalized by several centimeters—sometimes even shifted from one gyrus to another without passing through the intervening sulcus—thereby creating two spatially separated clusters from what is in reality a single localized source. By contrast, temporal imprecision is more tractable, and expression plots suggest that temporal error may be broadly Gaussian in nature. Motivated by this, we systematically evaluated a range of state-of-the-art temporal denoising techniques.

The hyperparameter configuration for each temporal denoiser was evaluated based on how well its results matched priors from literature. Therefore, we use the same configuration across transforms for interpretability of results; however, they could be further improved by leveraging transform-specific hyperparameters. In the following sections, when we describe the time complexity for each system, *N_H_*, is the number of hexels on the cortical surface (equal to 10,242 per hemisphere for our data), and *N_T_* is equal to the number of transforms.

The algorithm powering the clustering stage operates by preprocessing the expression plot, then running one of the temporal clustering algorithms below on the preprocessed data, and, finally, postprocessing the clustering output. The dimensions of the expression plot are latency on the *x*-axis and the surprisal [i.e., *-log(p)*] on the *y*-axis. Our preprocessing of these expression plots consists of removing insignificant expression points and discarding the surprisal dimension. Next, the selected clustering algorithm assigns each of the significant points to a cluster or tags them as an ‘anomaly’ (an expression point judged unlikely to belong to the IPPM). Finally, in postprocessing, we remove these anomalous points, tag the most significant point per cluster as the focus, and discard the rest of the points. The resulting expression plot contains only temporal foci, which can be used to draw an IPPM.

One could argue that it is better to select the temporal foci as the average centroid per cluster, rather than taking the most significant point. The primary advantage afforded by this strategy is that the average is more representative of the underlying cluster than the most significant point. Unfortunately, the issue with this approach is that the average is a virtual datapoint, i.e., it does not correspond to a real hexel and expression. Consequently, IPPMs, which are designed for interpretability, become more obscure. Moreover, the most significant point in a cluster is the point that displays the strongest evidence for a match. Therefore, it is the most likely location within a cluster where the transform appears.

#### 4.1.1 Max pooler

**Figure 5:**
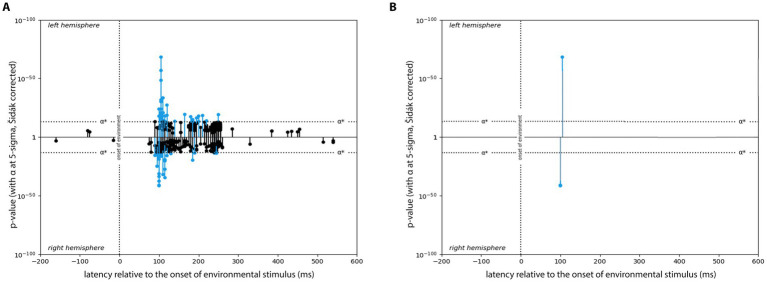
Illustration of ‘denoising’ expression data. **(A)** Original expression data for the transform ‘Heeger Horizontal velocity’. **(B)** The expression data for ‘Heeger Horizontal velocity’ following the clustering strategy of Max Pooling; the resulting map has been cleared of all spurious expression points.

The *Max Pooler* (MP) algorithm partitions the latency axis into fixed size bins and excludes bins lacking sufficient points to be a cluster. The bin size, *b*, and threshold for exclusion, *θ*, constitute the hyperparameters for MP. MP takes O(*N_T_ ·N_H_*) time to cluster because it requires one loop to assign each point to a bin, repeated for *N_T_* transforms. An example of this clustering is shown in Figure 5.

Like the other clustering algorithms, MP assumes certain properties that the clusters must satisfy. Specifically, it assumes that every cluster has the same size: *b.* Additionally, clusters must contain at least *θ* points, so high magnitude but isolated points are discarded as anomalies. Notably, for practical purposes, MP does not consider the surprisal of points.

MP, on the one hand, provides easy-to-interpret results, has low implementation overhead, and is not resource intensive. On the other hand, it makes inflexible assumptions about the nature of the clusters, particularly regarding cluster size.

#### 4.1.2 Adaptive max pooler

*Adaptive Max Pooler* (AMP) is an extension of MP that relaxes the assumption that each cluster has the same width. AMP takes the output of MP and merges adjacent clusters recursively, resulting in variable-width clusters. Instead of a fixed bin size, AMP uses a minimum bin width *b*; otherwise, AMP uses the same heuristics as MP.

Although there is an additional time penalty for looping through each bin and merging each bin, the time complexity remains O(*N_T_ ·N_H_*), the same as MP. Hence, AMP achieves greater generalization power with virtually no increase in complexity.

AMP is a “Goldilocks” solution: it is a well-rounded algorithm that balances adaptability and efficiency. However, its performance is sensitive to the choice of minimum bin width: setting it too high risks merging distinct clusters, while a value that is too low may fragment single clusters into multiple parts, particularly in cases with minor variations in the data.

#### 4.1.3 Gaussian mixture model

*Gaussian Mixture Modeling* (GMM) (Pearson, 1984; Dempster et al., 1977) is a generative machine learning approach that models expression plots as multimodal Gaussian distributions. The optimization is performed using the *Expectation–Maximization* (EM) algorithm (Dempster et al., 1977), which is commonly used in the context of latent variables. To determine the optimal number of Gaussians, our algorithm conducts a grid-search evaluated by AIC (Akaike, 1973). Additionally, GMM suffers from the *singularity* problem, the case where it fits a single Gaussian to a lone datapoint, leading to a singular covariance matrix and infinite likelihood. To mitigate this, GMM checks for singular covariance matrices after each fitting and reruns the fitting until it finds a non-singular matrix or reaches a maximum number of retries.

The time complexity for GMM is O(*N_T_*·*N_K_ ·I·T· N_H_ ·K·D*^2^), where *N_K_* is the number of Gaussians to grid-search up to, *I* is the number of initializations attempted, *T* is the maximum number of iterations, *K* is the number of clusters, and *D* is the number of dimensions. The *N_Ḥ_K·D*^2^ arises from the M-step, which dominates the EM algorithm’s time complexity due to the computation of the covariance matrix.

GMM assumes the underlying expression plot can be modeled by a multimodal Gaussian distribution, a reasonable assumption given the Gaussian blurring. While AIC performs best with large datasets due to its susceptibility to overfitting with smaller ones, using GMMs with fewer components helps address this by skewing the data-to-parameter ratio.

Due to the unconstrained covariance matrices, GMM can model elliptical clusters, making it more flexible than MP or AMP. Yet, such flexibility comes at the expense of computational complexity as GMM must be run numerous times per transform. Although, in practice, by keeping various hyperparameters, such as *I*, bounded, the computational overhead can be managed effectively.

#### 4.1.4 Mean shift

*Mean Shift* (Comaniciu and Meer, 2002) is a non-parametric, unsupervised machine learning algorithm, which models the expression plot using Kernel Density Estimation (KDE) (Rosenblatt, 1956; Parzen, 1962). Critically, Mean Shift does not require the number of clusters to be predetermined; it begins with the maximum number of clusters—one for each point—and merges nearby clusters by exploiting the Capture Theorem (Bertsekas, 2016). The time complexity for Mean Shift is O(*N_T_ ·T·N_H_*^2^), where *T* is the maximum number of iterations.

Mean Shift is the most general algorithm encountered so far, as it imposes no *a priori* assumptions about the nature of the underlying ground-truth, enabling it to model an arbitrary number of clusters and shapes. However, the performance of mean shift is heavily dependent on the *bandwidth* hyperparameter, which defines the initial cluster radius. As the radius of the clusters increases, the fitting becomes increasingly smoother but also more prone to underfitting. Moreover, using the same bandwidth globally implies the feature space is homogeneous, which is plausible given the Gaussian blurring. Finally, Mean Shift computes the distance between kernels in each iteration, leading to quadratic complexity with respect to dataset size, making it the most computational prohibitive algorithm.

#### 4.1.5 Density-based spatial clustering of applications with noise

*DBSCAN* (Ester et al., 1996) is a density-based, hierarchical clustering algorithm. It locates clusters by identifying core points, defined as points with at least *a set number of* points in an *ε*-neighborhood, and then attempting to jump to nearby points from these core points. Every point that is reachable from a core point, regardless of whether that is one or multiple jumps, is associated as part of that cluster. The time complexity for DBSCAN is (*N_T_ · N_H_ · log N_H_*).

DBSCAN uses density as a proxy for cluster plausibility and the same *ε* globally. Its generalization performance is akin to Mean Shift, as it places no assumptions on the shape or number of clusters, while also having slightly better time complexity. Furthermore, DBSCAN can be viewed as the next-generation approach to AMP. The primary difference between the two is that DBSCAN incorporates insignificant bins as part of a cluster if they are reachable from a significant bin. Consequently, it can overcome the fragmented cluster problem that affected AMP. Note that while DBSCAN is termed a “spatial clusterer,” in this work we use it to cluster in the temporal dimension.

### 4.2 IPPM builder

The *IPPM builder* constructs a DAG from the clustered expression map and the CTL. The CTL is provided as a dictionary, where child transforms are the keys, and each key is associated with a list of its parent transforms. The builder iterates through the transforms, starting with those that have no children. For each iteration, an edge is added from the final node of the parent transform to the initial node of the child transform. This loop continues, with the childless transforms being removed in each iteration, until no transforms remain, at which point the process terminates (see SI for more details). The final parent edges are connected to an input stream node, which has a latency of zero.

While this approach is robust enough to handle the scenarios illustrated in Figure 4, more complex cases involving intricate serial and parallel processes may require advanced DAG construction strategies. Future work could explore such strategies in greater detail.

## 5 Results

To evaluate the effectiveness of the temporal denoising approaches, we applied them to both simulated and experimental datasets. The simulated data provided a controlled setting with a known ground truth, enabling direct assessment of reconstruction accuracy, though it could not capture the full range of EMEG measurement errors. In contrast, the experimental data incorporated real-world noise and artifacts, but the true underlying signals were unknown. By combining insights from both simulated and experimental analyses, we aimed to obtain a more comprehensive evaluation of the proposed approaches.

### 5.1 Evaluation using simulated data

To examine the behavior of the IPPM-generation procedure—and specifically its ability to recover known effects under conditions of reduced signal strength—we first conducted a controlled simulation study.^1^

#### 5.1.1 Simulation methods

**Figure 6:**
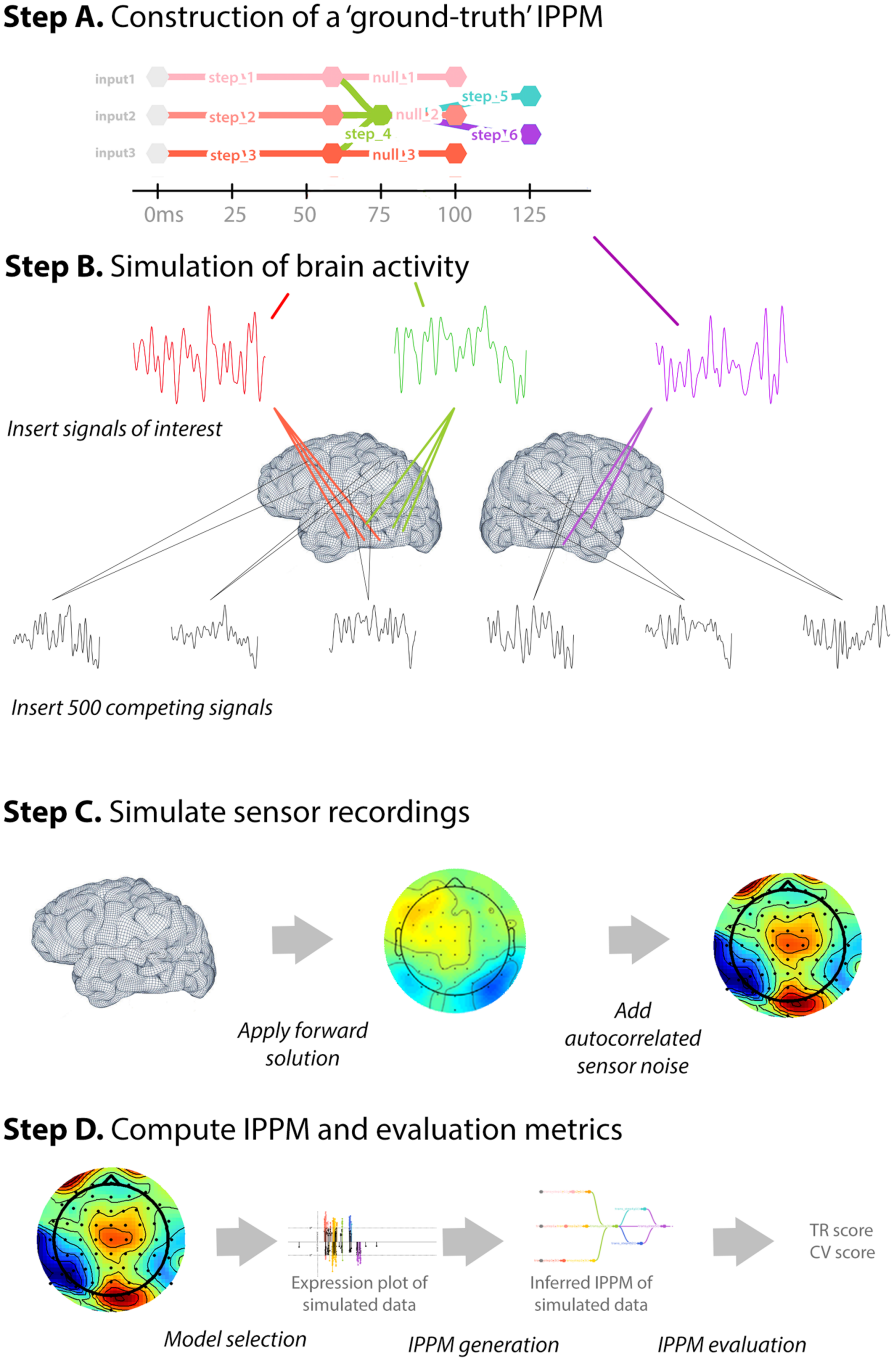
Simulation procedure. In (Step A) A ground-truth IPPM is produced. (Step B) Ground-truth brain activity simulated: synthetic computational signals from transforms in the source IPPM are inserted into bilateral ST and MT areas, and competing signals inserted into other bilateral areas. (Step C) Simulation of noisy sensor recordings. (Step D) Generation and evaluation of the derived IPPM.

We began with a predefined ‘ground truth’ IPPM, consisting of a (fictional) sequence of transforms with computational signatures assumed to exist in the brain, along with a corresponding input–output dependency graph (see Figure 6A). The ground truth IPPM was designed to result in potential CV and TR errors when noisy - it has three distinct input streams, each of which led to a distinct initial set of transforms expressed at 60 ms latency (*step_{1,2,3}*), then to both a set of null transforms (*null_{1,2,3}*) at 100 ms and combining into a single node (*step_4*) at 70 ms. From the output node of the *step_4* transform, two parallel transforms (*step_{5,6}*) are performed, with the output at 125 ms.

For each “ground truth” transform, we generated a synthetic computational signal by applying a 5–100 Hz band-pass filter to white noise. We used a single participant’s brain MR structural from Yang et al. (2025) and the cortical activity simulation functionality of MNE (Gramfort et al., 2013) to simulate synthetic cortical activity, inserted the synthetic computational signals at their respective latencies, at each of 10 single-hexel sources randomly selected from bilateral superior and middle temporal areas (Figure 6B) —latencies for individual sources were randomly jittered with a Gaussian offset (*σ* = 10 ms). To simulate competing activity, we inserted 500 further random synthetic computational signals (10 for each of 50 similarly band-passed random signals with uniformly random latencies ranging from 0 ms to 500 ms, similarly jittered) into the brain at sources randomly selected over the cortex (excluding bilateral superior and middle-temporal areas, so as not to overwrite the sources of interest, and also excluding the corpus callosum) with a maximum amplitude of 10 nAm. We used these synthetic sources to generate 30 s of simulated brain activity. Using MNE we applied the forward solution to this brain activity to compute the simulated electromagnetic field at the sensor locations of the scanner used by Yang et al. (2025). To this, we added simulated measurement noise (using MNE’s default parameters) using the sensor covariance matrix (Figure 6C). IPPMs may be generated from either source-localized data, or sensor recordings. To conserve computational resources, we elected to use the simulated sensor data rather than applying the inverse solution before beginning the Thwaites et al. (2017) model-selection procedure. We ran the simulation repeatedly, with the maximum amplitude of the “ground truth” sources of interest ranging from 0 to 10 nAm in steps of 0.5 nAm (we describe this in the results as signal strength *relative* to the competing sources, with values ranging from 0 to 100%). At each step, we simulated the noisy sensory data, applied the model-selection procedure to produce an expression plot, and then tested the five IPPM-generation methods in an attempt to recover the ground-truth IPPM (Figure 6D). We compared the recovered IPPM with the ground-truth IPPM using the CV and TR metrics.

#### 5.1.2 Simulation evaluation

**Figure 7:**
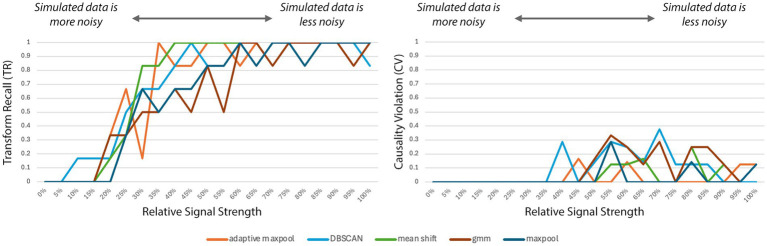
Left: Transform Recall (TR) as a function of increasing relative signal strength of the simulated ground-truth sources across all five IPPM-generation strategies. TR rises steadily as higher signal strength makes the correct IPPM easier to infer. Right: Causality Violation (CV) under the same conditions. CV is 0.0 when the data are too noisy to recover the IPPM, increases slightly as inference becomes possible, and then declines as signal strength improves.

Figure 7 illustrates how Transform Recall (TR) and Causality Violation (CV) vary with changes in the relative signal strength of the simulated ground-truth sources across all five IPPM-inference strategies. As expected, when the simulated sources had a magnitude of zero, no IPPM could be recovered, resulting in minimal TR and CV values of 0.0. With increasing signal strength, TR rose consistently across all methods, reflecting improved ability to correctly identify the underlying transforms. CV followed a different trajectory: it initially increased as the IPPM became just recoverable under noisy conditions, before decreasing again as higher signal strength allowed for more accurate inference of the true IPPM structure.

#### 5.1.3 Simulation evaluation discussion

The simulation study demonstrates that the automatic IPPM-generation procedure can successfully recover a defined ‘ground truth’ IPPM even when the target signals are heavily obscured by noise. At the lowest signal strengths (≤10%), no meaningful reconstruction was possible, but most ground-truth transforms were recovered once signal strength reached 40%, as shown by high transform recall at this point. Interestingly, even at high signal strengths (>70%), Transform Recall (TR) did not always reach 1.0, occasionally dropping to 0.9, This variability is likely due to re-randomization of source localizations and temporal jitter across simulation runs. Causality Violation (CV) starts to improve around 70%, although this likely reflects a finding specific to this simulation, due to the deliberate inclusion of challenging nodes in the ground-truth IPPM to test the robustness of the approaches. Nonetheless, the overall trend is clear: higher signal strength consistently leads to more accurate IPPM reconstruction, regardless of inference strategy.

These simulations are neither comprehensive nor fully realistic, as they omit several important sources of variability, such as anatomical differences between individuals, spatial autocorrelation of cortical sources, and variation in IPPM topology. Time and computational constraints further limited the complexity of the model. Future work should expand these simulations to systematically evaluate how IPPM-generation methods perform under different forms of noise, source locations, and network structures, particularly when investigating subtle differences between equipment, experimental conditions, or clinical populations (see Thwaites et al., 2025).

In real neuroimaging studies, the true underlying network is unknown and hypotheses must be informed by prior literature. However, these findings provide proof of principle: when a ground truth is defined, the proposed procedure can reliably recover it. This supports the conclusion that IPPMs can be reconstructed in practice, provided the CTL is accurate and the signal-to-noise ratio is sufficient.

### 5.2 Evaluation using experimental data

#### 5.2.1 Experimental data methods

For the above simulation experiments, the IPPM-generation approaches were applied with a range of reasonable default hyperparameter values. Because we had complete control over both the source data and the generation process in the simulation study, we deliberately avoided tuning hyperparameters on the simulated data to prevent overfitting to simulation-specific artifacts. To provide a more realistic evaluation, each automatic IPPM-generation strategy was further tested on two ‘real’ experimental expression datasets: one focused on loudness processing (Thwaites et al., 2017) and the other on horizontal motion processing (Wingfield et al., 2025). In the Thwaites et al. (2017) study, human loudness processing is hypothesized to comprise of 11 transforms which characterize the loudness of the auditory environment. In Wingfield et al. (2025), motion processing is modeled as six transforms. These two processing streams were chosen due to their well-studied and self-contained nature. IPPMs related to both processing streams were inferred from the publicly available Kymata SOTO Dataset (Yang et al., 2025). In this dataset, 15 participants underwent electroencephalography (EEG) and magnetoencephalography (MEG) recordings while listening to a podcast and watching colored dots move horizontally on a screen. The source dendritic currents estimated from these recordings were projected onto an average cortical surface. These 15 source maps were then averaged to create a single representative participant, from which the IPPMs were derived. Further methodological details are provided in Yang et al. (2025).

#### 5.2.2 Experimental data evaluation

All strategies had their hyperparameters tuned, optimizing for CV and TR. The first result of interest is that this hyperparameter tuning caused all the strategies to converge on a clustering solution characterized by global max pooling. This resulted in all the approaches giving the same result – these are shown in in Table 1.

**Table 1:** The TR and CV for both the loudness and motion IPPMs.

Loudness IPPM evaluation			Motion IPPM evaluation		
Metric name	Hemisphere	Metric score	Metric name	Hemisphere	Metric score
TR	Left	1	TR	Left	1
	Right	1		Right	1
	Both	1		Both	1
CV	Left	0	CV	Left	0
	Right	0.353		Right	0.111
	Both	0		Both	0.111

The evaluation for the left, right and both hemispheres are shown. All strategies converged on the same result, so only one result is shown here.

**Figure 8:**
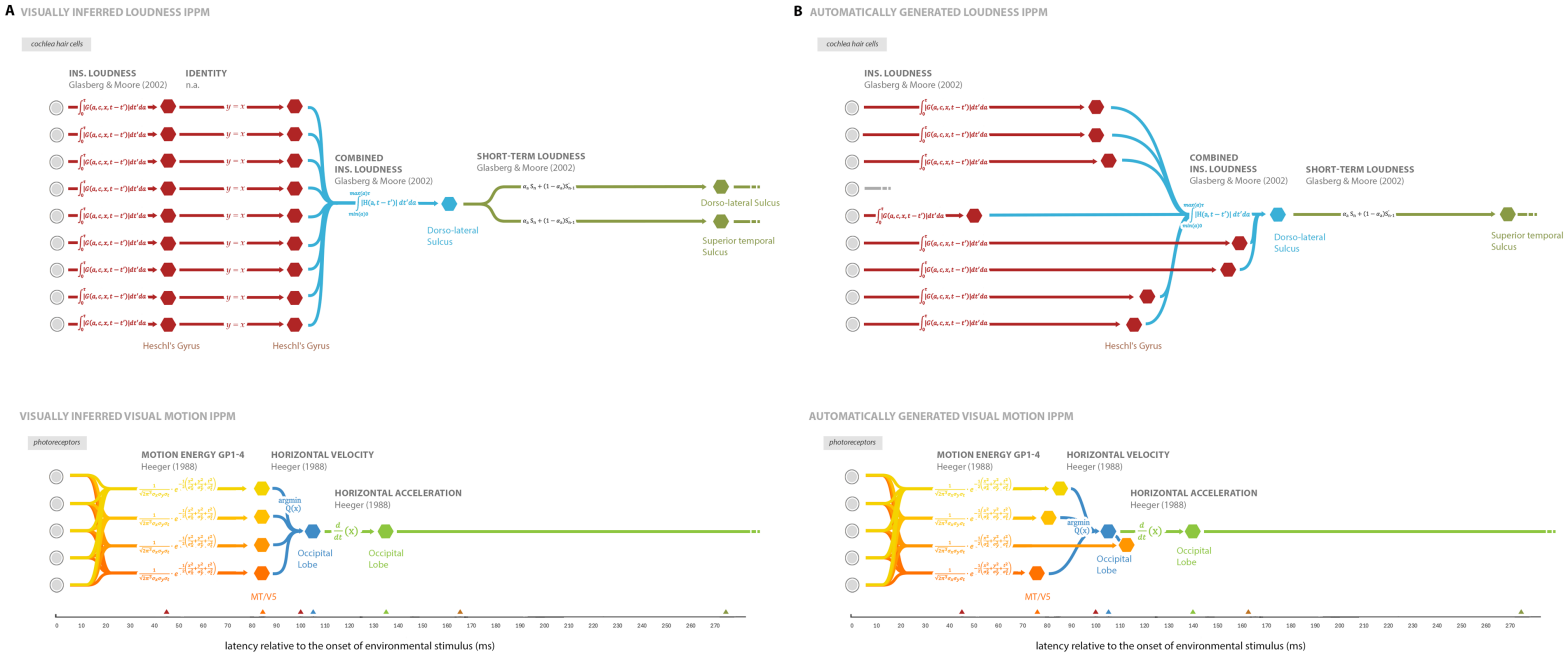
Comparison of the **(A)** IPPMs inferred through visual inspection of the expression plots (top) loudness processing (reproduced from Thwaites et al., 2017), and (bottom) motion processing (reproduced from Wingfield et al., 2025), and **(B)** their automatically generated counterparts. In both cases, the expression plot for each hemisphere was merged first, then denoised.

As a visual aid, we also print out a comparison of the visually inferred IPPMs reproduced from both Thwaites et al. (2017) and Wingfield et al. (2025), and their automatically generated counterparts (Figure 8). The automatically generated versions are relatively close to the hand versions, although there are some obvious differences, particularly in the loudness IPPM, where the identity transforms (where x = y), are missing, together with some of other nodes and edges. Potential reasons for this are covered in the discussion.

#### 5.2.3 Experimental data discussion

##### 5.2.3.1 Analysis of clustering strategies

After fine-tuning several temporal clustering strategies and running a hyperparameter grid search, we found that all strategies converged to a global max-pooling approach with respect to our evaluation metrics. While unexpected, this convergence can likely be explained by the tendency of the evaluation metrics to reward strategies that restrict each transformation to a single node. More specifically, TR is maximized after processing one node, so there is no benefit for the algorithm to incorporate additional clusters, and the addition of new nodes will likely deteriorate CV. As a result, both CV and TR pressure the generators to limit themselves to one node. This explains why the identity transforms are missing – multiple nodes for a transform are connected by null edges, representing the identity transform.

Although global max pooling optimizes both evaluation metrics, visual inspection of the expression maps do heavily imply that there should be multiple nodes loudness processing. To improve on this in future work one option would be to define an augmented TR: for example, GMMs can be performed prior to clustering, to identify the number of clusters, and an augmented TR could be defined as the average ratio of nodes to clusters over all transforms. This augmented TR would likely result in the retention of the identity transforms.

##### 5.2.3.2 Analysis of resultant IPPMs

All the automatic generation strategies performed poorly in the right hemisphere (see Supplementary materials). Thwaites et al. (2017) and Wingfield et al. (2025) contain stimuli which were presented monaurally and monocularly respectively, so both assumed symmetric processing across hemispheres. Therefore, the IPPMs were expected to be consistent. The cause of this poorer right-hemisphere performance is unclear, but there are some possibilities. It may reflect measurement error (as both datasets were acquired using the same EMEG equipment) or a low signal-to-noise ratio that introduces reconstruction errors. The simulation results support the latter interpretation, showing that at least one of the metrics (TR) decreases as signal strength declines, consistent with the reduced signal-to-noise ratio inherent in EEG/MEG source estimation. It is likely that in experimental data, the CV metric is also heavily influenced by signal strength. It is also possible that the assumption of symmetric bi-hemispheric processing is incorrect: the stimulus in the experiment was speech, and it is well documented that some language function is left-lateralised (e.g., Vikingstad et al., 2000), so even though the models here are for pre-linguistic transforms, it is possible that there is a natural lateralization to support downstream linguistic processes (cf. Wingfield et al., 2017, another EMEG study showing lateralized early responses to audio language stimuli).

The motion IPPM has higher CV than that of loudness, suggesting that it’s construction may be of slightly lower quality. However, it is important to note that CV is not suited for comparing IPPMs with different CTLs because different CTLs have a differing number of total edges, leading to different denominators. Analysis of the stem plots and IPPMs reveals this is caused by the most significant Heeger Horizontal ME GP3 node appearing after Heeger Horizontal Velocity. In the left hemisphere, the motion IPPM displayed no CV, implying that it is feasible to construct a no CV motion IPPM with the given data if different metrics were adopted.

One notable point is the absence of TVL loudness channel 4 from the loudness IPPM (marked as gray edges in Figure 6B). Since the TR is maximized, this implies that the underlying expression data did not contain any significant points for channel 4. Indeed Thwaites et al. (2017) note that it is missing in their own analysis, but they include this transforms in their visually inferred IPPM anyway; this underpins the importance of the current work removing such subjectivity from the IPPM generation procedure.

##### 5.2.3.3 Challenges

A fundamental challenge with the methodology described here is the reliance on the same data for both training and testing, due to the small amount of data available. As a result, the clustering algorithms overfit, inflating the evaluating metric scores, and the ability of the approach to generalize across diverse circumstances is not known. This problem should be overcome as an increasing number of expression datasets become available.

A second challenge lies in the assumption that both the CTL and the expression data are ‘correct’. This assumption may not be true - transforms in the CTL are, by their nature, approximations of human sensory processing, and expression data is derived from relatively noisy EMEG data. Over time, it is likely that the accuracy of both will be improved, and this will affect the accuracy of the resultant IPPMs that are generated.

There are three main ways we could improve the results: enriching the dataset quality, modifying the evaluation metrics, and modifying the clustering strategy.

###### 5.2.3.3.1 Improving the dataset

Improvements in the accuracy of the CTLs and expression maps would create narrower clusters, with higher peaks. This would make identifying distinct clusters easier.

Augmenting the expression data with the spatial location of each hexel may also improve performance. For example, the spatial proximity of two blurred clusters may hold valuable information that makes it easier to decide whether those blurred clusters are part of a single entity or disparate clusters. However, implementing this improvement is not trivial. Due to point spread error, expression clusters are spread across the folded cortical surface in ways that are rarely contiguous.

###### 5.2.3.3.2 Modifying the evaluation metrics

There is likely scope to improve the evaluation metrics used here. As mentioned above, TR could be modified to better measure the amount of salient information retained by the clustering algorithm, particularly the modality of the number of clusters for a transform.

More generally, there may be aspects of ‘good’ IPPMs that are not adequately covered by TR and CV. One is the complex interplay between serial and parallel processing, especially when confronted with multiple nodes. A discussion on what these more advanced metrics might look like is beyond the scope of this work, but it may require a more nuanced view from the research community as to what ‘good’ IPPMs should look like.

###### 5.2.3.3.3 Improving the clustering strategies

This study assumed that each transform exhibits the same pattern of noise, allowing the same set of hyperparameters to be applied for all transforms and leading to a transform-agnostic strategy. Hyperparameters could be set independently for each transform, but this is likely to yield only a modest improvement in performance at the cost of interpretability. Therefore, transform-agnostic strategies are preferred, even though they may result in slightly lower performance.

More generally however, considering all the cluster strategies converged on a single strategy, it seems likely that more example expression maps and CTLs will be a prerequisite to improving on their accuracy.

## 6 Conclusion

In this paper we have presented a procedure which automates the process of building IPPM graphs from cortical expression maps in a data-driven, objective manner. Furthermore, we have presented two evaluation metrics by which researchers can compare competing methods for building IPPM graphs.

In testing a range of possible strategies for clustering expression maps, it was found that all approaches converged on a global max pooling strategy. However, this is likely due to a lack of data, and there may be many improvements that can be made to improve IPPM creation in the future.

IPPMs are a representation of cortical processing, and the ability for researchers working in neuroimaging to create them in a manner free of subjectivity is an important challenge in the field. This paper hopes to have provided an outline of some of the tools and metrics that will be needed for this task.

## Data Availability

Publicly available datasets were analyzed in this study. This data can be found at: https://kymata.org/api/datasets/8.

## Footnotes

^1^We thank an anonymous reviewer for suggesting a simulation study.

## Authors note

Anirudh Lakra is now at Amazon.com, Inc., and his position at Amazon is unaffiliated with this work.
