# Supplementary material for: Strategies for automatic generation of information processing pathway maps
Source: Front Neuroimaging. 2025 Nov 25;4:1608390. doi: 10.3389/fnimg.2025.1608390 (PMC12687749; doi:10.3389/fnimg.2025.1608390)
Supplement: Supplementary file 1 [file Data_Sheet_1.docx]

**Supplementary information**

**Pareto Frontier for hyperparameter tuning**


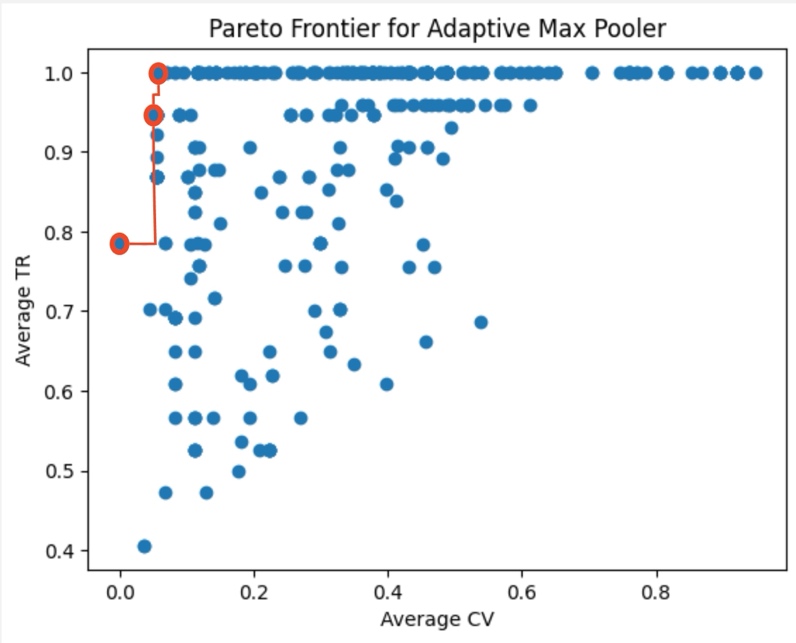


Figure SI1. The average TR and CV across hemispheres and datasets for different hyperparameter configurations, optimised for experimental data. The red dots indicate settings of TR and CV that lie on the Pareto Frontier: the set of points where you cannot optimize one metric without deteriorating the performance of the other metric. To choose between these points, the one that maximises TR at a satisfactory CV was chosen.

**IPPM Builder**

The IPPM builder class takes the clustered expression plot and CTL as inputs and outputs a DAG. The CTL is passed as a dictionary containing transforms as keys and a list of parent transforms as the value. As such, the CTL contains all the information we need to define the set of nodes and edges. From the clustered expression plot, we retrieve the node size, which is proportional to the magnitude, and the location on the latency axis.

The builder algorithm iterates through the transforms, isolates the expression data for the selected transform, constructs nodes for each central temporal foci effect, and, finally, draws edges to any children transforms.

A top-level transform is defined as a transform that does not transmit information to other transforms, i.e., it is childless. The builder iterates through the transforms in a top-down fashion. To add an edge, we require knowledge of the final node for the parent and the initial node for the child. By generating the graph top-down, we initialize the information for the child before the parent, so when we create the parent, we have all the information we need to add an edge from the last parent node to the first child node. The builder continues this loop and removes top-level transforms in each iteration; eventually, there will be no more transforms left, so it will terminate.

Input streams are a special case of transforms since we do not observe any expression data for them. However, we know that the stimulus begins at latency = 0, so we can construct a node in the IPPM at latency = 0.

The time complexity is O(*n_T_ * n_H_ log n_H_  + n_T_^2^*). The *n_H_ log n_H_* comes from sorting the central foci effects by latency, so we can rapidly add an edge from the last point of a parent to the first point of the child. Currently, the first term dominates the time complexity since *n_H_* is about 10,024. As the number of transforms increases, specifically past 10,024, we expect the *n_T_^2^* to dominate.

**Per-Hemisphere IPPMs**

Figure SI2 and SI3 shows the loudness and motion IPPMs respectively, split by hemisphere. The left-hemisphere IPPM showed substantially better reconstruction; possible reasons for this are discussed in Section 6.2.3.2.


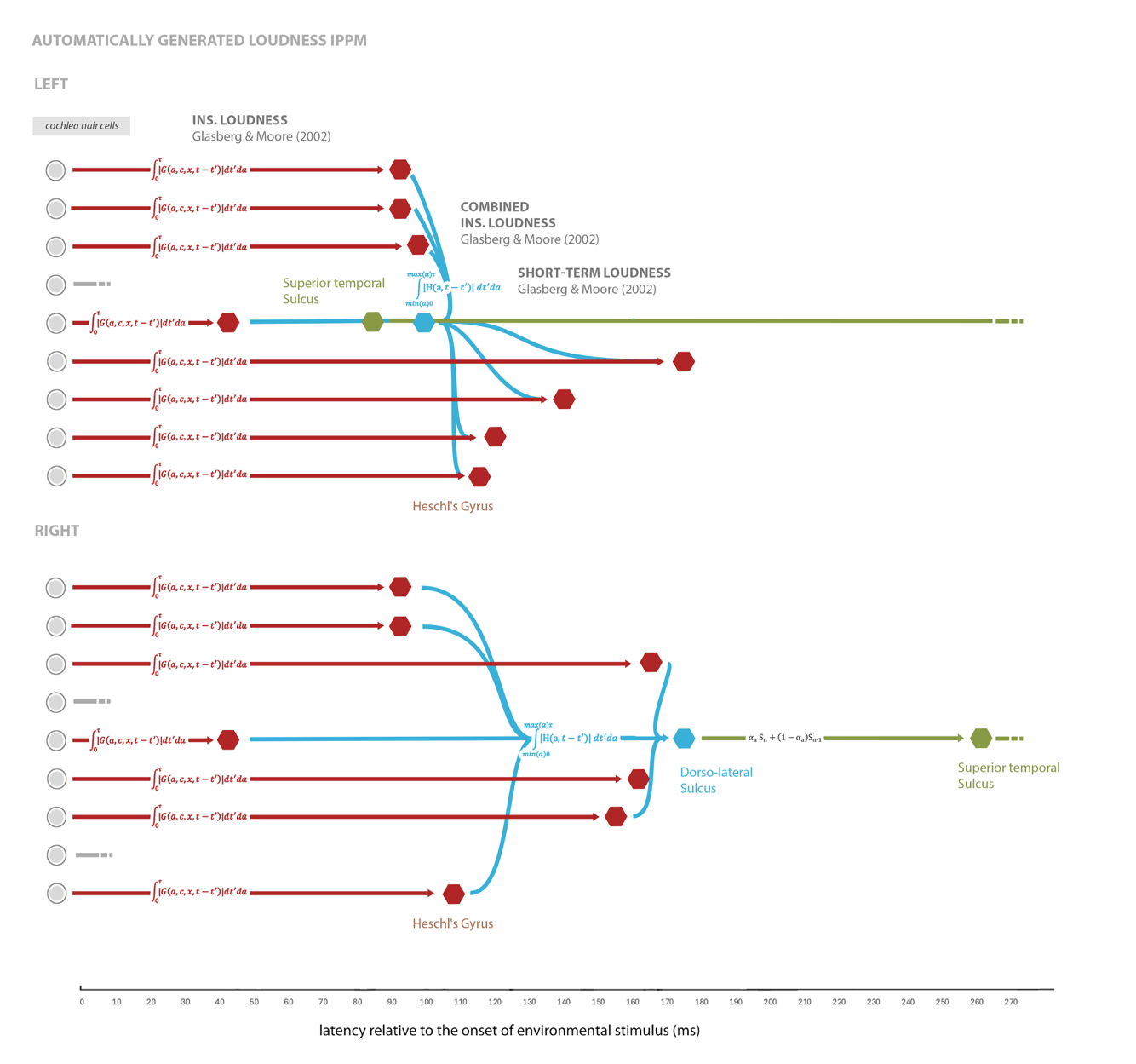


**Figure SI2. The loudness IPPM, when applied separately on each hemisphere.** The right hemisphere performs worse than the left hemisphere due to the large degree of CV introduced by TVL loudness (short-term) appearing before multiple child nodes. However, the right hemisphere has one advantage: it contains entrainment to TVL loudness channel 9. Consequently, the merged IPPM also contains channel 9 and selects the TVL loudness (short-term) node from the left hemisphere because it displays stronger evidence for a match, which resolves the CV.


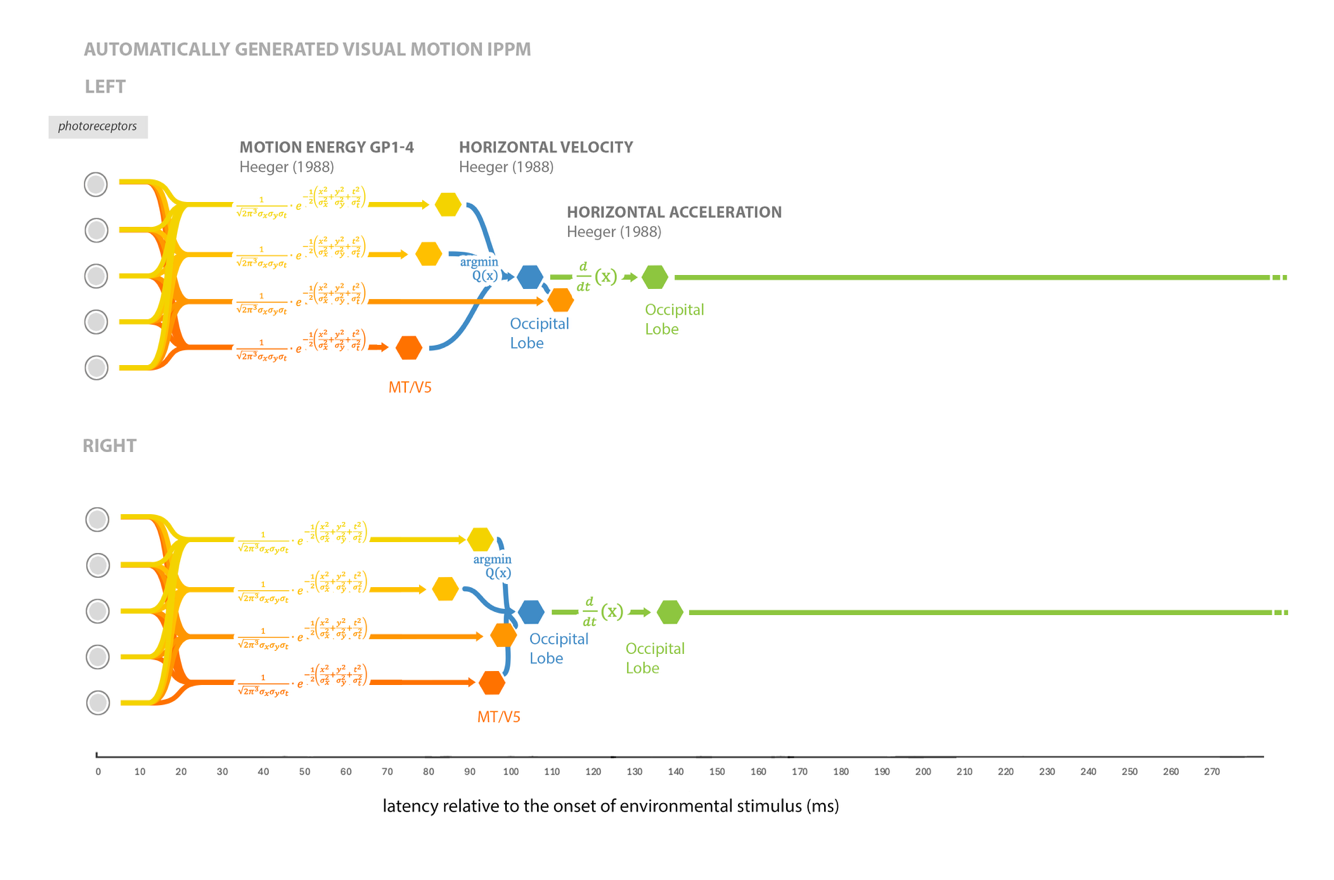


**Figure SI3. The motion IPPMs, when applied separately on each hemisphere.** Similarly to the loudness IPPMs, the right hemisphere displays additional CV. Both IPPMs contain the full list of transforms in the CTL, but the node for Heeger Horizontal ME GP3 in the right hemisphere is more significant than the node in the left, so it is chosen to be in the merged IPPM. Consequently, the merged IPPM also contains CV.
